# Loneliness, depression, anxiety, and post-traumatic stress disorder among Chinese adults during COVID-19: A cross-sectional online survey

**DOI:** 10.1371/journal.pone.0259012

**Published:** 2021-10-21

**Authors:** Zijun Xu, Dexing Zhang, Dong Xu, Xue Li, Yao Jie Xie, Wen Sun, Eric Kam-pui Lee, Benjamin Hon-kei Yip, Shuiyuan Xiao, Samuel Yueng-shan Wong

**Affiliations:** 1 JC School of Public Health and Primary Care, The Chinese University of Hong Kong, Hong Kong, China; 2 ACACIA Labs, School of Health Management and Institute for Global Health, Southern Medical University, Guangzhou, China; 3 Department of Pharmacology and Pharmacy, Li Ka Shing Faculty of Medicine, The University of Hong Kong, Hong Kong, China; 4 Department of Medicine, Li Ka Shing Faculty of Medicine, The University of Hong Kong, Hong Kong, China; 5 School of Nursing, The Hong Kong Polytechnic University, Hong Kong, China; 6 Xiangya School of Public Health, Central South University, Changsha, China; The University of Hong Kong, HONG KONG

## Abstract

**Objectives:**

This study aims to investigate the potential factors associated with mental health outcomes among Chinese adults during the Coronavirus disease 2019 (COVID-19) epidemic.

**Methods:**

This is an online cross-sectional survey conducted among Chinese adults in February 2020. Outcome measurements included the three-item UCLA Loneliness Scale (UCLA-3), two-item Patient Health Questionnaire (PHQ-2), two-item Generalized Anxiety Disorder Questionnaire (GAD-2), and two items from the Clinician-Administered Post-traumatic Stress Disorder (PTSD) Scale. COVID-19 related factors, physical health, lifestyle, and self-efficacy were also measured. Univariable and multivariable logistic regressions were performed.

**Results:**

This study included 1456 participants (age: 33.8±10.5 years; female: 59.1%). The prevalence of depressive symptoms, anxiety symptoms, loneliness, and PTSD symptoms were 11.3%, 7.6%, 38.7%, and 33.9%, respectively. In multivariable analysis, loneliness was associated with being single, separated/divorced/widowed, low level of education, current location, medication, more somatic symptoms, lower self-efficacy, and going out frequently. Depression was associated with fear of infection, binge drinking, more somatic symptoms, lower self-efficacy, and longer screen time. Anxiety was associated with more somatic symptoms and lower self-efficacy. PTSD symptoms were associated with more somatic symptoms, lower self-efficacy, higher perceived risk of infection, fear of infection, and self-rated more negative influence due to the epidemic (*p*<0.05).

**Conclusions:**

Mental health problems during the COVID-19 epidemic were associated with various biopsychosocial and COVID-19 related factors. Psychological interventions should be aware of these influencing factors and prioritize support for those people at higher risk.

## Introduction

In early December 2019, a confirmed case of Coronavirus disease 2019 (COVID-19) was reported in Wuhan, the capital city of Hubei province in China [1]. With a large crowd flow during the Spring Festival period in China, the number of COVID-19 cases increased rapidly. On 26^th^ February 2020, there were over 78,000 cases in China, which included more than 65,000 cases in Hubei province [2]. On 11^th^ March 2020, the World Health Organization (WHO) declared COVID-19 a worldwide pandemic [3].

The Chinese government had taken strict and effective public health measures to control the spread of the epidemic at the earliest time. Wuhan, where the epidemic was most serious, has been on lockdown since 23^rd^ January [4]. On 26^th^ January 2020, 30 provinces or cities in China had announced the launch of the first-level public health emergency response [4]. These safety measures included the cancelation of mass gatherings, limitation of transportation capacity, and the postponement of the spring semester [4]. Residents were suggested to stay home, maintain social distancing, wear protective face masks, and wash hands frequently. The government updated infection information to the public daily.

The unexpected COVID-19 epidemic, as well as the strict measures against the epidemic, may threaten people’s mental health. Behavioral Immune System (BIS) theory suggests that negative emotions, such as depressive and anxiety symptoms, and avoidance behaviors, including avoidance of human contacts or avoiding relevant information or activities, would appear for self-protection from external dangers [5, 6]. Some studies have been conducted to examine the level of mental health problems during the early epidemic in China. A nationwide survey indicated a mean COVID-19 Peri-traumatic Distress Index (CPDI) score of 23.65, and nearly 35% of respondents had experienced psychological distress [7]. Another study reported the prevalence of depression, anxiety, or the combination of the two were 48.3%, 22.6%, and 19.4%, respectively [8]. A web-based survey found an overall prevalence of anxiety symptoms, depressive symptoms, and poor sleep quality of 34.0%, 18.1%, and 18.1%, respectively [9]. Globally, the prevalence of depression and anxiety was 20% and 35%, respectively, during the COVID-19 outbreak [10].

Apart from the above-mentioned mental health problems, the unexpected epidemic and corresponding safety measures such as social distancing and home quarantine may also bring significant loneliness to the residents. During the epidemic of Severe Acute Respiratory Syndrome (SARS) in 2003, up to 38.5% of the people who experienced quarantine reported feelings of loneliness [11]. However, relatively few studies measured the prevalence of loneliness during COVID-19 in the general population. An online survey revealed that up to 47% of Chinese adults believed they may feel lonely for most of 2020 [12]. Overall, loneliness and other mental health problems may cause a significant burden to both individuals and society. As loneliness is an independent risk factor for many chronic diseases such as cardiovascular diseases, obesity, and mortality [13].

Given the high prevalence and potential health burden of mental health problems, targeted policies and interventions are urgently needed for the prevention and treatment during COVID-19 as well as for future readiness in unexpected pandemics or disasters. Therefore, understanding the risk factors associated with loneliness and other mental health problems is important. As researchers can design more targeted interventions by understanding and ameliorating these possible modifiable risk factors for improvement during this special time. Previous studies have found that many factors are associated with loneliness and other psychological symptoms, including physical health factors (e.g. chronic illness), social-cultural factors (e.g. social support, family, and marriage), and social environmental factors (e.g. rural versus urban environments) [14]. Although previous studies have examined the prevalence of depression and anxiety during COVID-19 [7, 9, 15], few studies have extensively examined the risk factors, especially COVID-19 related factors and self-efficacy, which are very likely to be associated with loneliness, depression, anxiety, and post-traumatic stress disorder (PTSD).

Therefore, we conducted this study to investigate these possible risk factors associated with loneliness, depressive symptoms, anxiety symptoms, and PTSD symptoms among Chinese adults one month after the closure of Wuhan city during COVID-19.

## Materials and methods

### Study design and study population

This is a cross-sectional study during the COVID-19 epidemic in China. The data were collected from 21-26^th^ February 2020. The target population comprised of Chinese adults aged 18 or above and were reached using convenience and snowball sampling methods. The questionnaire was developed on the platform of Wenjuanxing (www.wjx.cn). The investigators distributed the online survey link via Wechat, one of the most popular mobile applications for instant messages in China. The survey required about 10–15 minutes to complete and was completely voluntary and anonymous. The informed consent was provided at the beginning of the questionnaire (S1 File). After finishing the survey, all the participants would have received a report regarding their physical and mental health with information on help-seeking hotlines in case of need, and a lucky draw of 1–10 RMB. The study was approved by the Survey and Behavioural Research Ethics Committee of The Chinese University of Hong Kong and had been registered in a World Health Organization recognized registry (Registration No.: ChiCTR2000030223) before commencement.

### Measurements

The detailed measurements and data can be found in S1 and S2 Files.

#### COVID-19 related factors

COVID-19 related factors included taking part in anti-epidemic related work by oneself or family members, having confirmed or suspected cases among oneself, family members, acquaintances, and nearby residents in the city, perceived risk of being infected, fear of being infected, the perceived time needed for infection control, and overall self-rated influence due to COVID-19 epidemic.

#### Mental health outcomes

The primary outcome was loneliness, measured by a three-item UCLA Loneliness Scale (UCLA-3) [16]. A cut-off score of ≥4 represented a high level of loneliness [17]. Secondary outcomes included depressive symptoms, anxiety symptoms, and PTSD. Depressive symptoms were measured using a two-item Patient Health Questionnaire (PHQ-2), with a cut-off score of ≥3 considered positive [18]. Anxiety symptoms were assessed using a two-item Generalized Anxiety Disorder Questionnaire (GAD-2) [19]. A cut-off point of ≥3 was considered positive. PTSD symptoms were assessed by two questions about recurrent dreams and their avoidance, which were extracted from the Clinician-Administered post-traumatic stress disorder (PTSD) Scale [20]. A summation score of ≥3 signifies the existence of PTSD symptoms.

#### Self-efficacy

Self-efficacy, measuring the confidence to deal with unexpected events, was measured by one item from the General Self-Efficacy Scale; possible scores ranged from 1–4 with a higher score signifying a higher level of self-efficacy [21].

#### Physical health

The number of chronic diseases and the number of regular medications were self-reported. Somatic symptoms were measured by the validated 15-item Patient Health Questionnaire (PHQ-15) [22]. Self-reported overall physical health was rated from 1 = poor to 5 = excellent. Higher scores denote severer somatic symptoms.

#### Lifestyle

Smoking was recorded as current smoker, former smoker, and never smoker. Drinking was measured using one item in AUDIT-3, asking the frequency of binge drinking in the past year [23]. The exercise was the total hours doing physical exercise in the past week. Sedentary time was the average total hours of sitting or lying per day when awake in the past week. Screen time was the average daily use time of the mobile phone, Internet, TV, and video games in the past two weeks. The frequency of going outdoor and the distance walked in the past two weeks were also recorded.

### Statistical analysis

The demographic characteristics were described as frequency and percentage, or mean and standard deviation (SD). Logistic regressions were conducted to explore the potential risk factors influencing depressive and anxiety symptoms, loneliness, and PTSD. The associations between factors and loneliness, depressive symptoms, anxiety symptoms, and PTSD were demonstrated by the odds ratios (ORs), adjusted odds ratios (AORs), and their corresponding 95% confidence intervals (95%CIs). A two-tailed *p*-value less than 0.05 was considered statistically significant. All statistical analysis was performed using Stata version 13.1 (StataCorp. 2013. Stata Statistical Software: Release 13. College Station, TX: StataCorp LP.).

## Results

### Participant characteristics

A total of 1456 adults completed the online survey. The demographic data of participants is presented in Table 1. Most of our participants were female (59.1%), married (59.6%), employed (69.7%), living in the urban area (78.7%), and had at least a bachelor’s degree (73.3%). The mean age of the participants was 33.8±10.5 years old.

**Table 1:** Baseline characteristics (n = 1456).

Characteristics	Number (n)	Percentage (%)
Gender		
Male	596	40.9
Female	860	59.1
Age (mean±SD)	33.8±10.5	
Marriage		
Married	867	59.6
Single	552	37.9
Separated/divorce/widowed	37	2.5
Education		
Primary school and below	7	0.5
Middle school	64	4.4
High school	114	7.8
College degree	219	15.0
Bachelor degree	659	45.3
Postgraduate or above	393	27.0
Job		
Employed	1015	69.7
Unemployed	112	7.7
Student	305	20.9
Unknown	24	1.6
Income level		
Highest	17	1.2
Quite high	81	5.6
High	160	11.0
Average	874	60.0
Low	248	17.0
Quite low	47	3.2
Lowest	29	2.0
Current residence		
Rural	310	21.3
Urban	1146	78.7
Past-year residence		
Rural	172	11.8
Urban	1284	88.2
Current location		
Other provinces	1277	87.7
Hubei	114	7.8
Overseas	65	4.5

### Factors associated with mental health outcomes

#### Loneliness (UCLA-3)

About 38.7% (n = 563) of the participants were screened positive for loneliness. In univariable logistic regression, the UCLA-3 score was significantly lower in people with older age, higher education, better self-rated health, and higher self-efficacy (Table 2, p<0.05). The UCLA-3 score was significantly higher in unmarried people, students, binge drinkers, people with more chronic diseases, more medications, higher PHQ-15, having infected or suspected COVID-19 cases around, perceiving higher infection risk, longer sedentary and screen time, perceived longer time needed for infection control, fear of being infected, and reported being negatively influenced by the epidemic (Table 2, *p*<0.05).

**Table 2:** Univariable logistic regression analysis of factors influencing mental health outcomes.

Variables	Crude OR (95%CI)			
	Depression (PHQ-2)	Anxiety (GAD-2)	Loneliness (UCLA-3: 4–9)	PTSD (3–10)
Age	0.968 (0.951, 0.985)***	0.952 (0.931, 0.974)***	0.980 (0.970, 0.991)***	0.984 (0.973, 0.994)**
Gender (Female)	1.075 (0.772, 1.497)	0.852 (0.576, 1.261)	1.018 (0.821, 1.261)	0.906 (0.727, 1.129)
Marriage				
Married	ref	ref	ref	ref
Single	2.151 (1.546, 2.993)***	2.258 (1.518, 3.360)***	1.792 (1.439, 2.231)***	1.381 (1.105, 1.726)**
Separated/divorced/widowed	0.974 (0.292, 3.250)	1.020 (0.238, 4.372)	2.402 (1.239, 4.657)**	0.338 (0.130, 0.877)*
Education	1.082 (0.926, 1.264)	1.060 (0.881, 1.275)	0.883 (0.801, 0.972)*	1.013 (0.916, 1.120)
Job				
Employed	ref	ref	ref	ref
Unemployed	1.463 (0.817, 1.621)	1.106 (0.517, 2.368)	1.189 (0.799, 1.770)	1.006 (0.664, 1.523)
Student	1.900 (1.317, 2.741)**	1.864 (1.211, 2.870)**	1.355 (1.046, 1.757)*	1.269 (0.973, 1.654)
Unknown	0.860 (0.199, 3.714)	0.625 (0.083, 4.710)	0.703 (0.289, 1.710)	0.680 (0.267, 1.728)
Income	1.010 (0.853, 1.197)	1.140 (0.931, 1.396)	0.932 (0.834, 1.041)	1.013 (0.905, 1.135)
Current residence (Urban)	1.005 (0.676, 1.494)	0.913 (0.574, 1.454)	0.870 (0.674, 1.123)	0.950 (0.730, 1.237)
Past-year residence (Urban)	1.106 (0.659, 1.856)	0.573 (0.343, 0.957)*	0.837 (0.606, 1.156)	1.011 (0.722, 1.415)
Current location				
Other provinces	ref	ref	ref	ref
Hubei	1.942 (1.170, 3.229)*	2.047 (1.141, 3.673)*	0.973 (0.655, 1.444)	1.361 (0.920, 2.014)
Overseas	1.752 (0.894, 3.434)	1.631 (0.723, 3.679)	1.377 (0.835, 2.271)	1.178 (0.702, 1.975)
Smoking#	0.646 (0.365, 1.145)	1.162 (0.658, 2.053)	1.054 (0.764, 1.455)	1.262 (0.912, 1.747)
Binge drinking in the past year	2.272 (1.607, 3.214)***	2.346 (1.559, 3.531)***	1.710 (1.328, 2.201)***	1.628 (1.260, 2.104)***
Chronic disease	2.245 (1.609, 3.132)***	1.112 (1.027, 1.204)**	1.102 (1.030, 1.178)**	1.116 (1.042, 1.195)**
Medication	1.431 (1.133, 1.807)**	1.736 (1.352, 2.228)***	1.647 (1.368, 1.983)***	1.471 (1.226, 1.763)***
PHQ-15	1.229 (1.185, 1.274)***	1.262 (1.210, 1.315)***	1.217 (1.179, 1.255)***	1.164 (1.131, 1.198)***
Self-rated health	0.620 (0.516, 0.744)***	0.657 (0.529, 0.816)***	0.699 (0.622, 0.785)***	0.794 (0.706, 0.893)***
Self-efficacy	0.422 (0.334, 0.533)***	0.441 (0.335, 0.580)***	0.460 (0.389, 0.542)***	0.593 (0.505, 0.697)***
Anti-epidemic related work	1.060 (0.750, 1.498)	1.156 (0.767, 1.740)	1.043 (0.831, 1.308)	1.377 (1.094, 1.734)**
Cases around	2.302 (1.536, 3.451)***	1.867 (1.137, 3.064)*	1.677 (1.227, 2.293)**	1.827 (1.333, 2.503)***
Cases in the city	1.035 (0.947, 1.130)	0.994 (0.893, 1.205)	1.000 (0.944, 1.059)	1.040 (0.980, 1.103)
Perceived risk of being infected	1.327 (1.085, 1.623)**	1.689 (1.340, 2.128)***	1.402 (1.221, 1.611)***	1.602 (1.388, 1.850)***
Exercise	1.003 (0.998, 1.007)	0.993 (0.961, 1.026)	1.002 (0.998, 1.006)	0.994 (0.979, 1.009)
Sedentary time	1.060 (1.028, 1.093)***	1.039 (1.000, 1.078)*	1.023 (1.002, 1.045)*	1.010 (0.989, 1.032)
Going out frequency	0.958 (0.875, 1.048)	0.868 (0.777, 0.970)*	1.045 (0.986, 1.209)	0.942 (0.887, 1.001)
Going out distance	0.936 (0.861, 1.016)	0.904 (0.817, 1.000)*	1.006 (0.954, 1.060)	0.977 (0.925, 1.032)
Screen time	1.253 (1.138, 1.378)***	1.110 (0.993, 1.241)	1.074 (1.011, 1.141)*	1.018 (0.957, 1.083)
Perceived time needed for infection control	1.358 (1.098, 1.680)**	1.444 (1.127, 1.851)**	1.297 (1.119, 1.504)**	1.388 (1.193, 1.616)***
Fear of being infected	1.709 (1.328, 2.200)***	1.929 (1.421, 2.620)***	1.434 (1.220, 1.687)***	1.763 (1.487, 2.091)***
Negative influence^	1.552 (1.064, 2.265)*	2.037 (1.249, 3.322)**	1.534 (1.215, 1.937)***	1.688 (1.322, 2.156)***

Anti-epidemic related work: taking part in anti-epidemic related work by oneself or family members; Binge drinking: AUDIT-3, if tried binge drinking in the past year; Cases around: having confirmed or suspected cases among oneself, family members, acquaintances; Cases in the city: 1) 0, 2) <10, 3) 10–49, 4) 50–99, 5) 100–199, 6)> = 200; Chronic disease: total number of chronic diseases; Exercise: the total hours doing physical exercise in the past week; Education: 1 = Primary school and below, 2 = Middle school, 3 = High school, 4 = College degree, 5 = Bachelor degree, 6 = Postgraduate or above; Fear of being infected: 1 = Not worried; 2 = Worried; 3 = Very worried; GAD-2: two-item Generalized Anxiety Disorder Questionnaire; Going out distance: 1)<100 meters, 2) 100–499 meters, 3) 500–999 meters, 4) 1000–1999 meters, 5) 2–4.99 kilometers, 6) 5–9.99 kilometers, 7) 10–49 kilometers, 8) >50 kilometers; Going out frequency: 1 = Never, 2 = Less than once a week, 3 = Once a week, 4 = Two to three times a week, 5 Four to five times a week, 6 = almost every day; Medication: total number of medications taken regularly; Negative influence: overall self-rated influence due to COVID-19 epidemic; Perceived time needed for infection control: perceived time for successful epidemic control, 1) 1–2 months, 2) 3–6 months, 3) 6–12 months, 4) 1–2 years, 5) >3 years; Perceived risk of being infected: 1 = Very low, 2 = Low, 3 = High, 4 = Very high; PHQ-2: two-item Patient Health Questionnaire; PHQ-15: 15-item Patient Health Questionnaire; PTSD: post-traumatic stress disorder; Screen time: the average daily use time of mobile phone, Internet, TV, and video games in the past two weeks; Sedentary time: the average total hours of sitting or lying per day when awake in the past week; Self-efficacy: No matter what happens to you, you can handle it easily, 1 = not at all, 2 = partly right, 3 = quite right, 4 = absolutely right; Self-rated health: 1 = Bad, 2 = Average, 3 = Good, 4 = Very good, 5 = Extremely good; UCLA-3: three-item UCLA Loneliness Scale.

*p < 0.05;

**p < 0.01;

*** *p* < 0.001;

# Never or quit smoking as reference group;

^ No or positive influence as the reference group.

In the multivariable regression (Table 3), higher UCLA-3 score was still associated with being single (OR = 1.891, 95%CI: 1.316–2.717) or separated/divorced/widowed (OR = 2.675, 95%CI: 1.284–5.569), more medications (OR = 1.372, 95%CI: 1.087–1.731), higher PHQ-15 score (OR = 1.176, 95%CI: 1.134–1.220), higher going out frequency (OR = 1.110, 95%CI: 1.016–1.214). Lower UCLA-3 was still associated with higher education (OR = 0.787, 95%CI: 0.687–0.901), current location in Hubei province (OR = 0.483, 95%CI: 0.288–0.809), and higher self-efficacy (OR = 0.568, 95%CI: 0.469–0.688).

**Table 3:** Significant risk factors influencing mental health outcomes in multiple logistic regression analysis.

Variables	AOR (95%CI)			
	Depression (PHQ-2)	Anxiety (GAD-2)	Loneliness (UCLA-3: 4–9)	PTSD (3–10)
Single#	1.635 (0.955, 2.798)	1.619 (0.844, 3.104)	1.891 (1.316, 2.717)**	1.350 (0.941, 1.937)
Separated/divorced/widowed#	0.854 (0.234, 3.121)	1.091 (0.220, 5.417)	2.675 (1.284, 5.569)**	0.309 (0.113, 0.847)*
Education	1.068 (0.868, 1.315)	1.176 (0.909, 1.522)	0.787 (0.687, 0.901)**	0.981 (0.857, 1.123)
Hubei	1.330 (0.678, 2.611)	1.008 (0.448, 2.266)	0.483 (0.288, 0.809)**	0.723 (0.442, 1.183)
Binge drinking in the past year	1.835 (1.188, 2.835)**	1.271 (0.748, 2.160)	1.210 (0.889, 1.647)	1.218 (0.899, 1.651)
Medication	0.966 (0.707, 1.320)	1.138 (0.804, 1.612)	1.372 (1.087, 1.731)**	1.098 (0.881, 1.369)
PHQ-15	1.188 (1.136, 1.242)***	1.226 (1.165, 1.290)***	1.176 (1.134, 1.220)***	1.128 (1.090, 1.167)***
Self-efficacy	0.551 (0.410, 0.740)***	0.665 (0.464, 0.953)*	0.568 (0.469, 0.688)***	0.767 (0.636, 0.924)**
Anti-epidemic related work	0.930 (0.606, 1.426)	0.978 (0.586, 1.634)	0.987 (0.753, 1.294)	1.339 (1.026, 1.749)*
Perceived risk of being infected	0.865 (0.663, 1.127)	1.088 (0.801, 1.476)	1.086 (0.910, 1.295)	1.197 (1.009, 1.420)*
Going out frequency	1.077 (0.934, 1.243)	0.922 (0.772, 1.100)	1.110 (1.016, 1.214)*	0.920 (0.842, 1.005)
Screen time	1.151 (1.017, 1.301)*	1.045 (0.904, 1.208)	0.990 (0.914, 1.073)	0.962 (0.888, 1.042)
Fear of being infected	1.442 (1.071, 1.942)*	1.392 (0.971, 1.994)	1.209 (0.994, 1.470)	1.433 (1.182, 1.737)***
Negative influence^	1.148 (0.740, 1.779)	1.718 (0.975, 3.027)	1.214 (0.923, 1.596)	1.456 (1.106, 1.918)**

Non-significant factors in the full model are not shown in the table. Anti-epidemic related work: taking part in anti-epidemic related work by oneself or family members; Binge drinking: AUDIT-3, if tried binge drinking in the past year; Education: 1 = Primary school and below, 2 = Middle school, 3 = High school, 4 = College degree, 5 = Bachelor degree, 6 = Postgraduate or above; Fear of being infected: 1 = Not worried; 2 = Worried; 3 = Very worried; GAD-2: two-item Generalized Anxiety Disorder Questionnaire; Going out frequency: 1 = Never, 2 = Less than once a week, 3 = Once a week, 4 = Two to three times a week, 5 Four to five times a week, 6 = almost every day; Medication: total number of medications taken regularly; Negative influence: overall self-rated influence due to COVID-19 epidemic; Perceived risk of being infected: 1 = Very low, 2 = Low, 3 = High, 4 = Very high; PHQ-2: two-item Patient Health Questionnaire; PHQ-15: 15-item Patient Health Questionnaire; PTSD: post-traumatic stress disorder; Screen time: the average daily use time of mobile phone, Internet, TV, and video games in the past two weeks; Self-efficacy: No matter what happens to you, you can handle it easily, 1 = not at all, 2 = partly right, 3 = quite right, 4 = absolutely right; Self-rated health: 1 = Bad, 2 = Average, 3 = Good, 4 = Very good, 5 = Extremely good; UCLA-3: three-item UCLA Loneliness Scale.

*p < 0.05;

**p < 0.01;

*** *p* < 0.001;

# Married as the reference group;

^ No or positive influence as the reference group.

#### Depressive symptoms (PHQ-2)

One-hundred and sixty-five participants (11.3%) scored ≥3 on PHQ-2, signifying the presence of significant depressive symptoms. In the univariable analysis (Table 2), an increased risk of depressive symptoms was significantly associated with being single, student, currently in Hubei province, binge drinking in the past year, having chronic diseases, more medications, higher PHQ-15 score, having confirmed or suspected cases around, high perceived risk of being infected, longer sedentary and screen time, perceived longer time needed for infection control, fear of being infected, and reported more negative influence due to COVID-19 (*p*<0.05). A decreased risk of depressive symptoms was significantly related to older age, higher self-efficacy, and better self-rated health (*p*<0.05).

In the multivariable logistic regression model (Table 3), an increased level of depressive symptoms was independently and significantly associated with binge drinking in the past year (OR = 1.835, 95%CI: 1.188–2.835), higher PHQ-15 score (OR = 1.188, 95%CI: 1.136–1.242), low self-efficacy (OR = 0.551, 95%CI: 0.410–0.740), longer screen time (OR = 1.151, 95%CI: 1.017–1.301), and fear of being infected (OR = 1.442, 95%CI: 1.071–1.942).

#### Anxiety symptoms (GAD-2)

One hundred and ten (7.6%) participants were screened positive for GAD-2. In the univariable analysis (Table 2), anxiety symptoms were associated with younger age, being single, being a student, past-year residence in a rural area, current location in Hubei province, binge drinking, more chronic diseases, more medications taken regularly, higher PHQ-15, lower self-rated health, lower self-efficacy, cases around, higher perceived risk of being infected, longer sedentary time, going out more frequently and farther, longer perceived control time, fear of being infected, and overall negative influence due to COVID-19 (*p*<0.05).

In the multivariable regression analysis (Table 3), anxiety was still significantly associated with higher PHQ-15 scores (OR = 1.226, 95%CI: 1.165–1.290) and worse self-efficacy (OR = 0.665, 95%CI: 0.464–0.953).

#### PTSD symptoms

A total of 494 (33.9%) participants had significant PTSD symptoms. In Table 2, many factors were related to PTSD symptoms, including younger age, single, separated/divorced/widowed, binge drinking in the past year, more chronic disease, more medication, higher PHQ-15, worse self-rated health, lower self-efficacy, anti-epidemic related work, cases around, higher risk of being infected, perceived longer time needed for infection control, more fear of being infected, and negative influence (*p*<0.05).

In multivariable regression (Table 3), separated/divorced/widowed (OR = 0.309, 95%CI: 0.113–0.847), PHQ-15 (OR = 1.128, 95%CI: 1.090–1.167), self-efficacy (OR = 0.767, 95%CI: 0.636–0.924), anti-epidemic related work (OR = 1.339, 95%CI: 1.026–1.749), perceived risk of being infected (OR = 1.197, 95%CI: 1.009–1.420), fear of being infected (OR = 1.433, 95%CI: 1.182–1.737), negative influence (OR = 1.456, 95%CI: 1.106–1.918) remained significant.

## Discussion

Although the public health measures had successfully slowed down the dissemination of the epidemic in China one month later after the outbreak, these strict measures might continue to affect the mental health of the residents. Our study explored the possible immediate mental health effects on loneliness, depressive and anxiety symptoms, and PTSD and possible associated factors from multiple dimensions during the peak of the COVID-19 outbreak in China. Our results showed that many factors were associated with loneliness, depressive symptoms, anxiety symptoms, and PTSD in this particular situation.

Fear of being infected, high perceived risk of being infected, the self-rated overall negative influence due to COVID-19, and taking part in anti-epidemic related work by oneself or family members were related to PTSD symptoms in our study. People may repeatedly think of or dream about the COVID-19, and avoid COVID-19 related events. PTSD is a common phenomenon during the outbreak of infectious diseases, which has been observed during the epidemic of SARS [24–26] and current studies in the COVID-19 epidemic [7, 27]. The prevalence of PTSD symptoms was over 30% in the current study. Among SARS survivors, PTSD symptoms were the most prevalent and would exist in the long term, as well as depressive symptoms [28]. Therefore, during and after the COVID-19 epidemic, more attention should be paid to residents’ mental health, especially PTSD symptoms.

High self-efficacy is found to be a protective factor for all mental health outcomes. This self-efficacy seemed also to play a role in protecting from worse mental health during COVID-19. Our findings were consistent with previous studies that low self-efficacy was a predictor of loneliness and psychological distress [29–31], and high self-efficacy may be an independent protective factor for loneliness and other mental health problems. The self-efficacy theory of depression explained the independent association between low self-efficacy and high risk of depression [32]. Low self-efficacy may affect mental health through the following ways: people may feel or believe that they are unable to achieve satisfying performance, develop satisfying relationships with others, and control disturbing depressive ruminations [32]. In our study, we mainly measured their confidence to deal efficiently with unexpected events. Its content was consistent with satisfying performance. Those people who showed higher self-efficacy in the unexpected COVID-19 outbreak might have more knowledge and resources in a difficult situation. Future studies might take a closer eye on people who might show higher self-efficacy and how to build up self-efficacy, in preparing people with readiness for future events and situations.

Somatic symptoms were a strong independent risk factor for loneliness and all other mental health outcomes in this study, including depressive and anxiety symptoms, and PTSD. Taking more regular medications was also associated with loneliness. However, other physical health factors were not associated with mental health outcomes. The relationship has been confirmed in previous studies that somatic symptoms were closely related to loneliness, depression, and anxiety before COVID-19 [33–36]. However, one of these studies proposed that somatic symptoms frequently originate from mental illness because somatic symptoms had little association with physical diseases [33]. Further analysis should confirm their causal relationship and explore if it was the epidemic that affected somatic symptoms, for example, people could have been more aware of body reactions in face of monitoring possible symptoms related to infection. On the other hand, in Chinese culture, people tend to somaticize mental health problems, meaning they express somatic symptoms instead of mental problems in clinical consultations [37, 38]. Health professionals may need to pay special attention to mental health problems when a client presents somatic symptoms without indicated impaired physical health.

The COVID-19 epidemic and the accompanying control measures had a certain impact on loneliness. Longitudinal studies indicated that there was a significant increase in loneliness after the COVID-19 outbreak [39]. In the current study, almost 40% of the participants reported loneliness during the COVID-19 epidemic. Loneliness may be relieved after the epidemic is controlled and mitigation measures are relaxed. However, if social isolation continues, attention and measures may be needed to reduce loneliness. In this study, people living in Hubei province were less likely to feel lonely. This may be mainly explained by the nationwide attention, support, and encouragement during the epidemic, through formal and informal online and offline channels, that many social forces were helping Hubei to control the epidemic with charitable donations, volunteer activities, and medical assistance [4]. This should have been a good example of how social support could mitigate loneliness during the pandemic, for people in the most affected areas. Overall, with the epidemic and infection control measures continue, mental health services should be provided to the general population affected by the COVID-19 epidemic [40, 41].

The prevalence of depressive and anxiety symptoms of 11.3% and 7.6% were lower than that reported in Huang’s study, where the prevalence of depression and anxiety were 18.1% and 34.0% [9]. Our study was conducted in late February, about two weeks after Huang’s study. The lower prevalence is probably because the epidemic in China was under control to some extent by then, or due to a large proportion of non-Hubei samples or more higher-educated people in our sample. A recent national survey found that a self-developed comprehensive index of mental health was significantly associated with younger age, female, higher education, occupation such as migrant workers, and middle region of China [7]. Another study found that higher trust in doctors, perceived survival possibility, low risk of infection, health information satisfaction, and personal preventive measures were the protective factors for poor mental health [42]. In our study, fear of being infected and more daily screen time in the past two weeks were related to depression symptoms. Many people were advised to stay at home or work online at home during the epidemic, thus increased the use of electronic products. A previous systematic review found that frequent mobile phone use was a risk factor for depressive symptoms among adult populations [43]. It was not clear why these people were more depressed, though it was likely due to more exposure to negative information on the internet, or being less active for other activities especially outdoor activities.

### Strengths and limitations

This study provided a comprehensive view of mental health problems during the COVID-19 epidemic. We also explored the risk factors for mental health problems from various aspects, such as COVID-19 related factors, self-efficacy, physical health, lifestyle, and demographic characteristics. The current study also has several limitations. First, this was an online survey using convenience and snowball sampling, which may hardly include people who do not have access to the internet. Although the results in this study may not be representative of the general population of Chinese adults, this should have not influenced our conclusions on the associated risk factors. Second, to ensure the study feasibility and response rate, we used PHQ-2, GAD-2, and part of the PTSD scale to screen depressive symptoms, anxiety symptoms, and PTSD symptoms instead of more accurate and detailed scales although some of these scales have been well validated [18, 19]. These were to avoid bringing too much burden to the respondents as the compressed questionnaire still took ten minutes to finish and may be longer for some others. However, PHQ-2 and GAD-2 showed high sensitivity and specificity in screening depression and anxiety, respectively [18, 44].

In summary, loneliness and other mental health problems during the COVID-19 epidemic in China were associated with many factors, including gender, marriage, location, binge drinking, medication, somatic symptoms, screen time, self-efficacy, COVID-19 related factors (including anti-epidemic related work, perceived risk of being infected, going out frequency, fear of being infected, and perceived overall COVID-19 influence). The results can help identify high-risk groups for poor mental health and promote the screening of mental health problems, as well as providing information for more targeted interventions when mental health resources are scarce.

## Supporting information

S1 File Original Chinese and translated English questionnaires and informed consent used in this study. (DOCX)

S2 File Data used in this study. (SAV)
