# Supplementary material for: Loneliness, depression, anxiety, and post-traumatic stress disorder among Chinese adults during COVID-19: A cross-sectional online survey
Source: PLoS One. 2021 Oct 21;16(10):e0259012. doi: 10.1371/journal.pone.0259012 (PMC8530321; doi:10.1371/journal.pone.0259012)
Supplement: S1 File — (DOCX) [file pone.0259012.s001.docx]

**中国居民新型冠状病毒传播期间生活质量和身心健康状况调查**

Survey of quality of life, physical and mental health among adults in China during COVID-19

尊敬的居民：

您好！

首先感谢您支持此项问卷调查工作。自2019年12月在我国湖北省武汉市爆发新型冠状病毒肺炎以来，全国各地均在采取严格的管理措施以控制疾病的扩散。在疾病控制期间，居民的生活质量和心理健康状况受到不同程度影响，为了解目前居民在特殊时期的身心健康，为采取必要的干预方案做参考和准备，因此该项问卷调查工作迫切需要得到您的支持、理解和配合。

该问卷包括个人的基本情况、新型冠状病毒有关的情况、焦虑、抑郁、生活质量、健康状况、就医行为等方面。

问卷调查以**不记名方式**进行，回答无所谓对错，结果只作为整体分析之用。在您决定自愿参加该项研究工作之后，请您按照自己的实际情况如实回答。

本问卷包括约60条问题，绝大多数为选择题，请您阅读题目要求，按照自己的实际情况回答。您回答的真实性和填写的完整性对于我们工作的准确性十分重要。

问卷提交后，您会获得一个**微信红包**（1元至10元不等），以及**一份属于您的身心健康报告**，请您保存。其中的健康建议仅供个人参考，并**不能完全代表专业诊断**。如果您觉得有帮助，请将此问卷转给您的亲朋好友，帮助更多的人了解自己的健康状况、获得健康报告和建议吧。


谢谢！
2020年2月

Dear resident,

Hello!

First of all, thank you for supporting this survey. Since the outbreak of COVID-19 in Wuhan, Hubei Province in December 2019, strict management measures have been taken throughout the country to control the spread of the disease. During the period of disease control, the quality of life and mental health of residents were affected to varying degrees. To understand the physical and mental health of residents in special periods and make references and preparation for taking necessary intervention plans, this survey needs your support, understanding, and cooperation.

The questionnaire included the demographic, COVID-19 related issues, anxiety, depression, quality of life, health status, medical seeking behavior, and so on.

The questionnaire was conducted **anonymously**. There is no correct answer and the results are only used for our overall analysis. After you decide to voluntarily participate in the research, please answer truthfully according to your actual situation.

The questionnaire includes about 60 questions, most of which are multiple-choice questions. Please read the questions and answer according to your actual situation. The authenticity of your answer and the completeness of your filling is very important to the accuracy of our work.

After submitting, you will receive a **Wechat lucky draw** (ranging from 1 to 10 RMB) and **a report on your physical and mental health**. Please keep it. The health recommendations are for personal reference only and **do not fully represent professional diagnosis**. If you think this is helpful, please kindly transfer this survey to your relatives and friends to help more people understand their health status and obtain reports and suggestions on their health.

Thank you!
2020 February

1. {q1_gender}您的性别? Gender

| ○1=男Male |
| --- |
| ○2=女Female |

2. {q2_age}您的年龄（周岁）? Age

_________________________________

3. {q3_marriage}您的婚姻? Marriage

| ○1=已婚Married |
| --- |
| ○2=未婚Singe |
| ○3=再婚Re-married |
| ○4=同居Cohabited |
| ○5=分居Separated |
| ○6=离婚Divorced |
| ○7=丧偶Widowed |

4. {q4_job}您目前的职业为? Job

| ○1=医务人员Medical staff |
| --- |
| ○2=医学生Medical student |
| ○3=农民/渔民Farmer/fisherman |
| ○4=工人/服务人员Worker/service staff |
| ○5=除医务人员以外的专业技术人员/行政管理人员Professional technical personnel/administrative management personnel other than medical personnel |
| ○6=个体户/临时工Self-employed/temporary worker |
| ○7=离退休/病休Retired |
| ○8=无业/失业Unemployed |
| ○9=除医学生以外的其他学生Students other than medical students |
| ○10=家务Housework |
| ○11=其他Others |

5. {q5_edu}您的文化程度是? Education

| ○1=小学及以下Primary school and below |
| --- |
| ○2=初中 Middle school |
| ○3=高中High school |
| ○4=大专College |
| ○5=本科Undergraduate |
| ○6=研究生及以上Postgraduate and above |

6. {q6}您目前人在哪里? Location

| ○1=湖北省武汉市 Wuhan, Hubei |
| --- |
| ○2=湖北省内、武汉市外的其他地区 Other cities in Hubei |
| ○3=湖北省以外的其他省市Other provinces |
| ○4=国外或海外 Overseas |

7.[矩阵单选题] *

|  | 农村Rural | 城市Urban |
| --- | --- | --- |
| 1) 您目前居住在  Current residence{q7_1}： | ○=1 | ○=2 |
| 2) 您过去一年大部分时候居住在residence in the past year{q7_2}： | ○=1 | ○=2 |

[Income is included as q9.]

9. {q9}您最近一年的家庭收入，在您平时生活的地方属于什么水平? What is the level of your family income in the last year where you usually live?

| ○1=最高Highest |
| --- |
| ○2=比较高Relatively high |
| ○3=偏高High |
| ○4=中等Average |
| ○5=偏低Low |
| ○6=比较低Relatively low |
| ○7=最低 Lowest |

[Cases in the city is included as q11.]

11. {q11} 您目前所在的城市是否已有确诊的新型冠状病毒肺炎患者? Is anyone in your city infected with COVID-19?

| ○1=无 No |
| --- |
| ○2=有，少于10例 Yes, less than 10 |
| ○3=有，10-49例 Yes, 10-49 |
| ○4=有，50-99例 Yes, 50-99 |
| ○5=有，100-199例 Yes, 100-199 |
| ○6=有，200例或以上 Yes, 200 or more |
| ○7=有，但不知道例数 Yes, but do not know the number |

[Cases around is included as q12, q13, and q14.]

12. {q12}您的家人或近亲是否有人感染了新型冠状病毒? Is anyone in your family or close relatives infected with COVID-19?

| ○1=有人确诊感染Yes |
| --- |
| ○2=有人疑似感染，但未确诊Suspected infection but not diagnosed |
| ○3=无人感染No |

13. {q13}您的同事或朋友是否有人感染了新型冠状病毒? Is anyone of your colleagues or friends infected with COVID-19?

| ○1=有人确诊感染Yes |
| --- |
| ○2=有人疑似感染，但未确诊Suspected infection but not diagnosed |
| ○3=无人感染或不知道No |

14. {q14}您所居住的小区/村寨是否有人感染了新型冠状病毒? Is anyone in your community infected with COVID-19?

| ○1=有人确诊感染Yes |
| --- |
| ○2=有人疑似感染，但未确诊Suspected infection but not diagnosed |
| ○3=无人感染或不知道No  [Anti-epidemic related work is included as q14.]  15. {q15}您或家人是否參加了前线抗疫工作?（如医护、警务人员，社区保安、环卫、后勤、运输等Are you or your family member take part in anti-epidemic work (e.g. medical workers, policemen, community securities, environmental sanitation workers, transportation workers)?   \| ○1=是，仅自己 \| \| --- \| \| ○2=是，仅家人 \| \| ○3=是，自己和家人 \| \| ○4=否 \| |

[Fear of being infected is included as q16.]

16. {q16}您是否担心自己或家人会被新型冠状病毒感染? Are you worried that you or your family will be infected by COVID-19?

| ○1=已确诊感染Infected |
| --- |
| ○2=非常担心Very worried |
| ○3=担心Worried |
| ○4=不担心Not worried |

[Perceived risk of being infected is included as q17.]

17. {q17}您觉得自己感染新型冠状病毒的可能性有多高? How likely do you think you will be infected by COVID-19?

| ○1=已确诊感染Infected |
| --- |
| ○2=非常高Very high |
| ○3=高High |
| ○4=低 Low |
| ○5=非常低 Very low |

[PHQ-15 is included as q19_1 to q19_15.]

19.过去四周，您是否受以下情况困扰? Over the last week, how often have you been bothered by the following?

1) {q19_1}胃/肚痛 Stomach pain

| ○1=没有困扰Not at all |
| --- |
| ○2=少许困扰Bothered a little |
| ○3=很多困扰Bothered a lot |

2) {q19_2}背痛 Back pain

| ○1=没有困扰Not at all |
| --- |
| ○2=少许困扰Bothered a little |
| ○3=很多困扰Bothered a lot |

3) {q19_3}胳膊、 腿或关节疼痛（膝、髋关节等）Pain in arms, legs or joints (knees, hips, etc.)

| ○1=没有困扰Not at all |
| --- |
| ○2=少许困扰Bothered a little |
| ○3=很多困扰Bothered a lot |

4) {q19_4}痛经或其他月经问题 Menstrual cramps or other problems you’re your periods

| ○1=没有困扰Not at all |
| --- |
| ○2=少许困扰Bothered a little |
| ○3=很多困扰Bothered a lot |
| ○-3=跳过Skip |

5) {q19_5}性生活中有疼痛或其他问题Pain or problems during sexual intercourse

| ○1=没有困扰Not at all |
| --- |
| ○2=少许困扰Bothered a little |
| ○3=很多困扰Bothered a lot |

6) {q19_6}头痛Headache

| ○1=没有困扰Not at all |
| --- |
| ○2=少许困扰Bothered a little |
| ○3=很多困扰Bothered a lot |

7) {q19_7}胸痛Chest pain

| ○1=没有困扰Not at all |
| --- |
| ○2=少许困扰Bothered a little |
| ○3=很多困扰Bothered a lot |

8) {q19_8}头晕Dizziness

| ○1=没有困扰Not at all |
| --- |
| ○2=少许困扰Bothered a little |
| ○3=很多困扰Bothered a lot |

9) {q19_9}短时间昏倒Fainting spells

| ○1=没有困扰Not at all |
| --- |
| ○2=少许困扰Bothered a little |
| ○3=很多困扰Bothered a lot |

10) {q19_10}感觉心跳加重或急速地跳动Feeling your heart pound or race

| ○1=没有困扰Not at all |
| --- |
| ○2=少许困扰Bothered a little |
| ○3=很多困扰Bothered a lot |

11) {q19_11}呼吸急促Shortness of breath

| ○1=没有困扰Not at all |
| --- |
| ○2=少许困扰Bothered a little |
| ○3=很多困扰Bothered a lot |

12) {q19_12}便秘、肠道不适、或腹泻Constipation, loose bowels or diarrhea

| ○1=没有困扰Not at all |
| --- |
| ○2=少许困扰Bothered a little |
| ○3=很多困扰Bothered a lot |

13) {q19_13}恶心、胀气、或消化不良nausea, has or indigestion

| ○1=没有困扰Not at all |
| --- |
| ○2=少许困扰Bothered a little |
| ○3=很多困扰Bothered a lot |

14) {q19_14}感到疲劳或精力不足Feeling tired or having low energy

| ○1=没有困扰Not at all |
| --- |
| ○2=少许困扰Bothered a little |
| ○3=很多困扰Bothered a lot |

15) {q19_15}睡眠有问题Trouble sleeping

| ○1=没有困扰Not at all |
| --- |
| ○2=少许困扰Bothered a little |
| ○3=很多困扰Bothered a lot |

21.下面列举了一些人们可能会有的情况，请选择适合您的答案Here are some situations that people may have, please choose the answer that suits you

[PHQ-2 is included as q21_1 and q21_2.]

1) {q21_1}近两周我做事提不起劲或没有兴趣 Little interest or pleasure in doing things in the past two weeks?

| ○1=没有Not at all |
| --- |
| ○2=有几天Several days |
| ○3=一半以上时间More than half the days |
| ○4=几乎每天Nearly every day |

2) {q21_2}近两周我感到情绪低落、沮丧或绝望 Feeling down, depressed, or hopeless in the past two weeks

| ○1=没有Not at all |
| --- |
| ○2=有几天Several days |
| ○3=一半以上时间More than half the days |
| ○4=几乎每天Nearly every day |

[GAD-2 is included as q22 and q23.]

22. {q22}近两周我感到不安、担心或烦躁 Feeling nervous, anxious, or on edge in the past two weeks

| ○1=没有Not at all |
| --- |
| ○2=有几天Several days |
| ○3=一半以上时间More than half the days |
| ○4=几乎每天Nearly every day |

23. {q23}近两周我不能停止担心或控制不了担心 Not being able to stop or control worrying in the past two weeks

| ○1=没有Not at all |
| --- |
| ○2=有几天Several days |
| ○3=一半以上时间More than half the days |
| ○4=几乎每天Nearly every day |

[PTSD is included as q24 and q25.]

24. {q24}您有没有反复不安地、控制不住地想到或梦到新冠肺炎有关的事件? Have you repeatedly thought about or dreamed about events related to COVID-19?

| ○1=没有Absent |
| --- |
| ○2=轻度Mild / subthreshold |
| ○3=中度Moderate / threshold |
| ○4=重度Severe / markedly elevated |
| ○5=极重Extreme / incapacitating |

25. {q25}您有没有回避与新冠肺炎有关的信息、人、活动、地点、想法、感觉等 Have you avoided information, people, activities, places, thoughts, and feelings related to COVID-19?

| ○1=没有Absent |
| --- |
| ○2=轻度Mild / subthreshold |
| ○3=中度Moderate / threshold |
| ○4=重度Severe / markedly elevated |
| ○5=极重Extreme / incapacitating |

[Loneliness is included as q26, q27, and q28.]

26. {q26}您是否觉得自己缺乏人陪伴? How often do you feel that you lack companionship?

| ○1=几乎没有Rarely |
| --- |
| ○2=有时Sometimes |
| ○3=经常Often |

27. {q27}您是否觉得被忽略? How often do you feel left out?

| ○1=几乎没有Rarely |
| --- |
| ○2=有时Sometimes |
| ○3=经常Often |

28. {q28}您是否觉得自己被其他人孤立? How often do you feel isolated from others?

| ○1=几乎没有Rarely |
| --- |
| ○2=有时Sometimes |
| ○3=经常Often |

[Self-efficacy is included as q31.]

31. {q31}无论什么事在您身上发生，您都能够应付自如。I can usually handle whatever comes my way

| ○1=完全不正确Not at all true |
| --- |
| ○2=小部分正确 Partly true |
| ○3=多数正确 Mostly true |
| ○4=完全正确Exactly true |

[Medication is included as q35.]

35. {q35}你现时需定期或持续服用多少种药物（只计医护人员处方的药物）? How many medications you need to take regularly (prescription)?

| ○1=无需服用 No |
| --- |
| ○2=服用1种 One |
| ○3=2-3种 Two to three |
| ○4=4种或以上 Four or more |

[Exercise is included as q42 and q43.]

42. 在过去七日，您做剧烈运动的时间总共有多长呢?（剧烈运动是指做完后，呼吸会非常急速，例如跑步、跳操、踢足球、游泳、做粗重工作等。）In the past seven days, how long did you spend doing vigorous exercise in total? (Vigorous exercise refers to the rapid breathing after you finish, such as running, aerobics, playing football, swimming, doing heavy work, etc.){q42}_________分钟 minutes

43. 在过去七日，您做中等强度的体能活动的时间总共有多长呢?（中等强度体能活动是指做完后，呼吸会比平常急速，例如踏单车、洗车打腊、快步走、擦窗等。）In the past seven days, how long did you spend doing moderate-intensity physical activities in total? (Moderate-intensity physical activity means that your breathing will be faster than usual after you do it, such as cycling, car washing and waxing, brisk walking, rubbing Windows etc.){q43}__________分钟 minutes

[Sedentary time is included as q44.]

44. 在过去七日，清醒的时候，您有多少时间是坐着或者躺着的呢? In the past seven days, when awake, how much time did you sit or lie down?
平均{q44}________小时/天  Average __________hours/day

[Smoking is included as q46.]

46. {q46}您是否吸烟?  Do you smoke?

| ○1=吸烟 Smoke |
| --- |
| ○2=从不吸烟 Never smoke |
| ○3=已戒烟 Quit smoke |

[Binge drinking in the past year is included as q48.]

48. {q48}过去一年，您试过一次饮至少5罐或5杯酒精饮品吗? （指任何类型的酒杯或罐的总数，而一次是指在几个小时之内。）During the past year, how often do you have five or more drinks on one occasion?

| ○1=没有试过 Never |
| --- |
| ○2=试过，少于每月一次 Less than once a month |
| ○3=试过，每月两次 Twice a month |
| ○4=试过，每月三次或以上 Three or more times a month |

[Going out frequency is included as q50.]

50. {q50}最近2周，您外出的频率是? In the past 2 weeks, how often did you go out?

| ○1=从未出门Never |
| --- |
| ○2=少于一周一次Less than once a week |
| ○3=一周1次Once a week |
| ○4=一周2-3次 Two to three times a week |
| ○5=一周4-5次Four to five times a week |
| ○6=几乎每天 Almost every day |

[Going out frequency is included as q51.]

51. {q51}最近2周，您的活动范围半径? What is the radius of your activity in the last 2 weeks?

| ○1= <100米 Less than 100 meters |
| --- |
| ○2= 100-499米 100-499 meters |
| ○3= 500-999米 500-999 meters |
| ○4= 1000-1999米 1000-1999 meters |
| ○5= 2公里-4.99公里 2-4.99 kilometers |
| ○6= 5公里-9.99公里 5-9.99 kilometers |
| ○7= 10公里-49公里 10-49 kilometers |
| ○8= 50公里及以上 More than 50 kilometers |

[Screen time is included as q52.]

52. {q52}最近2周，您平均每日的手机、网络、电视、游戏机的使用时间: In the last 2 weeks, your average daily usage time of mobile phones, internet, TV, and game consoles:

| ○1=无 No |
| --- |
| ○2=少于1小时 Less than an hour |
| ○3=1-2小时 1-2 hours |
| ○4=3-4小时 3-4 hours |
| ○5=5-6小时 5-6 hours |
| ○6=7-8小时7-8 hours |
| ○7= 9-10小时 9-10 hours |
| ○8=10小时以上 More than 10 hours |

[Negative influence is included as q58_14.]

14) {q58_14}整体而言，目前新冠肺炎疫情对您各方面的影响程度：Overall, the current degree of impact of the COVID-19 epidemic on you in all aspects:

| ○1=负面影响，非常大 Very negative impact |
| --- |
| ○2=负面影响，比较大Relatively large negative impact |
| ○3=负面影响，比较小Relatively small negative impact |
| ○4=无影响 No impact |
| ○5=正面影响，比较小Relatively small posotive impact |
| ○6=正面影响，比较大Relatively large positive impact |
| ○7=正面影响，非常大Very positive impact |

[Perceived time needed for infection control is included as q60.]

60. {q60}您估计要多少时间国家新型冠状病毒的疫情就可以被完全控制住? How long do you estimate that the country's COVID-19 outbreak can be completely controlled?

| ○1= 1-2个月 1-2 months |
| --- |
| ○2= 3-6个月 3-6 months |
| ○3= 半年-1年 Half to one year |
| ○4= 1-2年 1-2 years |
| ○5= 3年以上 More than 3 years |

[Chronic disease is included as q61_1 to q61_53.]

61. 您有没有曾被医生诊断有以下情况? (请选出所有适合的答案) Have you ever been diagnosed by a doctor with the following conditions（ Please choose all the appropriate answers)

| □{q61_1}高血压Hypertension | □{q61_2}心脏病（冠心病、心律失常、心脏瓣膜病等）Heart disease |
| --- | --- |
| □{q61_3}恶性肿瘤/癌症 Cancer {q61_3a}_________________* | □{q61_4}哮喘Asthma |
| □{q61_5}脂肪肝Fatty liver | □{q61_6}胃食管返流Gastroesophageal reflux |
| □{q61_7}慢性肠炎Chronic enteritis | □{q61_8}慢性疼痛(需药物控制) Chronic pain (drug control needed) |
| □{q61_9}高尿酸、痛风High uric acid, gout | □{q61_10}膀胱疾病Bladder diseases |
| □{q61_11}卵巢、输卵管异常Abnormal ovary and fallopian tube | □{q61_12}梅尼埃病Meniere's disease |
| □{q61_13}白内障Cataract | □{q61_14}湿疹Eczema |
| □{q61_15}偏头痛Migraine | □{q61_16}精神分裂症Schizophrenia |
| □{q61_17}焦虑症Anxiety disorder | □{q61_18}糖尿病Diabetes |
| □{q61_19}中风/脑血管疾病Stroke / cerebrovascular disease | □{q61_20}慢性阻塞性肺病Chronic obstructive pulmonary disease |
| □{q61_21}肺纤维化pulmonary fibrosis | □{q61_22}其他慢性肝病Other chronic liver diseases |
| □{q61_23}胃炎、胃溃疡Gastritis, gastric ulcer | □{q61_24}肠易激综合症Irritable bowel syndrome |
| □{q61_25}关节炎Arthritis | □{q61_26}甲状腺疾病Thyroid disease |
| □{q61_27}前列腺疾病Prostate disease | □{q61_28}鼻炎、鼻窦炎Rhinitis and sinusitis |
| □{q61_29}中耳炎Tympanitis | □{q61_30}其他眼部问题（失明，弱视、视网膜脱落、病变等）Other eye problems |
| □{q61_31}牛皮癣（银屑病）Psoriasis (psoriasis) | □{q61_32}癫痫Epilepsy |
| □{q61_33}双相障碍Bipolar disorder | □{q61_34}痴呆症Dementia |
| □{q61_35}血脂异常Dyslipidemia | □{q61_36}周围血管疾病Peripheral vascular diseases |
| □{q61_37}支气管扩张Bronchiectasia | □{q61_38}病毒性肝炎（乙肝，丙肝等）Viral hepatitis (hepatitis B, hepatitis C, etc.) |
| □{q61_39}胆囊疾病Gallbladder disease | □{q61_40}十二指肠溃疡Duodenal ulcer |
| □{q61_41}肠息肉Intestinal polyp | □{q61_42}骨质疏松Osteoporosis |
| □{q61_43}肾脏疾病（慢性肾病(肾炎)，肾结石等）Kidney disease | □{q61_44}子宫异常Abnormal uterus |
| □{q61_45}失聪/耳鸣Deafness / tinnitus | □{q61_46}青光眼Glaucoma |
| □{q61_47}红斑狼疮Lupus erythematosus | □{q61_48}血液疾病（贫血、血友病等）Blood diseases (anemia, hemophilia, etc.) |
| □{q61_49}帕金森病Parkinson's disease | □{q61_50}抑郁症Depression |
| □{q61_51}结核（肺或其他部位结核）Tuberculosis of lung or other parts | □{q61_52}其他疾病{q61_52a} Other diseases_________________* |
| □{q61_53}无任何疾病 None of above |  |

[Self-rated health is included as q62.]

62. {q62}总括来说，您认为您的健康状况是: In general, how would you rate your health

| ○1=极好Very good |
| --- |
| ○2=很好 Good |
| ○3=好 Moderate |
| ○4=一般 Bad |
| ○5=差 Very bad |
